# Specific downregulation of cystathionine *β*‐synthase expression in the kidney during obesity

**DOI:** 10.14814/phy2.13630

**Published:** 2018-07-12

**Authors:** Mi Liu, Mokan Deng, Jiahui Su, Yu Lin, Zhanjun Jia, Kexin Peng, Fei Wang, Tianxin Yang

**Affiliations:** ^1^ Institute of Hypertension Sun Yat‐Sen University School of Medicine Guangzhou China; ^2^ Department of Medicine and Veterans Affairs Medical Center University of Utah Salt Lake City Utah; ^3^ Department of Pathology Zhujiang Hospital Southern Medical University Guangzhou China; ^4^ Nanjing Key Laboratory of Pediatrics Nanjing China

**Keywords:** Cystathionine *β*‐synthase, cystathionine *γ*‐lyase, high fat, hydrogen sulfide, obesity

## Abstract

Hydrogen sulfide (H_2_S) is recognized as a novel gasotransmitter involved in the regulation of nervous system, cardiovascular functions, inflammatory response, gastrointestinal system, and renal function. Cystathionine *β*‐synthase (CBS) and cystathionine *γ*‐lyase (CSE) are the major enzymes responsible for H_2_S production through desulfuration reactions. H_2_S is reported to play a protective role in both high‐fat diet (HFD)‐induced obese and diabetic mice. However, the synthesizing enzyme involved is not clearly elucidated. The current study was aimed to investigate the regulation of CBS and CSE in different tissues including the kidney, liver, and epididymal fat in C57BL/6 mice after a HFD (60% kcal fat) for 24 weeks. The protein and mRNA expression of CBS was specifically decreased in the kidney while CSE remained unchanged, which was further confirmed in db/db mice. In the liver, CSE expression was downregulated after HFD accompanied with unchanged CBS. Moreover, CSE expression was even upregulated in epididymal fat. The specific downregulation of renal CBS may contribute to decreased H_2_S production, which could be a pathogenic mechanism of obesity. Increased CSE/H_2_S pathway in epididymal fat possibly resulted in impaired glucose uptake and aggravated insulin resistance. In conclusion, our results revealed that CBS was selectively downregulated in both diet and gene‐induced obesity models.

## Introduction

According to the World Health Organization updated January 2015, 39% of adults aged 18 years and over were overweight in 2014, of which 13% were obese. Imbalance between energy uptake and expenditure is the major reason of obesity. Overweight or obese individuals have an increased risk of kidney diseases compared to those with normal weight (Wang et al. 2008). Furthermore, except for an independent risk factor for chronic kidney diseases, obesity also attribute to aggravated course and poor outcomes of chronic kidney diseases even after adjustment for confounding comorbidities such as diabetes and hypertension (Eknoyan 2007). Since the increasing prevalence of obesity and resulting health problems, it is important to investigate the mechanism of obesity‐related diseases and identify potential targets to ameliorate the progression of diseases.

Hydrogen sulfide (H_2_S) is recognized as the third gasotransmitter besides nitric oxide (NO) and carbon monoxide (CO) and has attracted attention because of its pleiotropic physiological effects. Endogenous H_2_S raises largely from two pyridoxal phophate (PLP)‐dependent enzymes, cystathionine *β*‐synthase (CBS) and cystathionine *γ*‐lyase (CSE), and also from PLP‐independent 3‐mercaptopyruvate sulfurtransferase (3‐MST) (Kimura 2011). Recently Shibuya et al. (2013) discovered a novel pathway of H_2_S production which involved 3‐MST and D‐amino acid oxidase from D‐cysteine. CBS and CSE are widely but variably distributed in different tissues. Generally, CBS is a predominant source of H_2_S in the central nervous system while CSE in peripheral system. Additionally, in some tissues such as the kidney and liver, both enzymes contribute to H_2_S synthesis.

It has been reported that H_2_S treatment increased glucose uptake in both myotubes and adipocytes and ameliorated kidney lesions in type‐2 diabetes by increasing insulin sensitivity (Xue et al. 2013). Liu et al. (2014) showed the decreased plasma H_2_S levels in db/db mice and that H_2_S improved wound healing via restoration of endothelial progenitor cell (EPC) functions in type‐2 diabetes. However, renal H_2_S or CBS/CSE regulation in type‐2 diabetes remains unknown. On the other hand, there are some existing reports about H_2_S regulation in high‐fat diet‐induced obesity (DIO) models but none conclusive and consistent results have been yielded. Peh et al. (2014) reported decreased H_2_S production in the kidney and liver during high‐fat diet, which was accompanied with unchanged CBS and CSE protein levels in the kidney and increased CBS but decreased CSE protein in the liver. At the same time, another group showed increased mRNA and protein levels of both CBS and CSE in the liver after high‐fat diet (Hwang et al. 2013). As for CSE/H_2_S pathway in adipose tissues, the paradoxical regulation was shown in the same study that increased or decreased H_2_S had the same effect on ameliorating insulin resistance in high‐fat diet (HFD) induced obese mice (Geng et al. 2013). In the present study, firstly we employed DIO model to demonstrate tissue‐specific regulation of CBS and CSE. Secondly, we validated the results using db/db mice. Overall, we present a comprehensive investigation of regulation of H2S synthases in various tissues during metabolic stresses.

## Materials and Methods

### Animals and diet

Obese‐diabetic Lepr^db/db^ (db/db, B6.BKS (D)‐leprdb/J), Lepr^db/m^ (db/m, lean control), and C57/BL6 male mice were purchased from the Jackson Laboratories (Bar Harbor, ME). For the DIO model experiment, C57/BL6 mice aged 8 weeks were fed for 24 weeks with either a control diet (10% kcal fat, catalog No. 58124) or a high‐fat diet (HFD) (60% kcal fat, catalog No. 58126) (Test Diet, St. Louis, MO). The details about diet composition were shown in Table 1. Mice had free access to food and water. For fasting experiment, C57/BL6 mice aged 8 weeks were fed for 24 h with either enough control diet or no food, accompanied with free access to water. The db/m control and db/db mice also had free access to food and water. All the mice were kept on a 12 h (light): 12 h (dark) cycle. At the end of all experiments, animals were sacrificed and then the kidney, liver, heart and epididymal fat rapidly harvested. The tissues were frozen in liquid nitrogen or immersed in 4% neutral buffered formalin. Frozen tissues were stored at −80°C. All protocols employing mice were conducted in accordance with the principles and guidance of the Sun Yat‐sen University Institutional Animal Care and Use Committee.

**Table 1:** Composition of control diet (10% kcal fat) and high‐fat diet (60% kcal fat)

Macronutrients	Control diet	High‐fat diet
Protein	18.0%	18.1%
Carbohydrate	71.8%	20.3%
Fat source	10.2% (18 ppm cholesterol, 1.39% linoleic acid, 0.19% linolenic acid, 0.19% Omega‐3 fatty acid, 1.14% total saturated fatty acids and 1.3% total monounsaturated fatty acids)	61.6% (301 ppm cholesterol, 4.7% linoleic acid, 0.39% linoleic acid, 0.06% arachidonic acid, 0.39% Omega‐3 fatty acid, 13.68% total saturated fatty acids and 14% total monounsaturated fatty acids)

### Cystathionine *β*‐synthase activity assay

CBS activity was measured by the ninhydrin assay as previously described (Kriebitzsch et al. 2011). In brief, tissues were homogenized in isolation solution (containing 10 mmol/L triethanolamine and 250 mmol/L sucrose, pH 7.6) with protease inhibitor cocktail (Roche). The supernatant was aspirated after centrifugation at 12,000 rpm for 15 min at 4°C. For the assay, 100 *μ*L sample supernatant, 10 *μ*L Tris‐HCl (1 mol/L), 2 *μ*L pyridoxal phosphate (1.2 mmol/L in 0.1 mol/L Tris, pH 8.3) (Sigma‐Aldrich), and 20 *μ*L propargylglycine (25 mmol/L in 0.1 mol/L Tris, pH 8.3) (Sigma‐Aldrich) were mixed and incubated for 15 min at 37°C. Subsequently, 20 *μ*L serine (1 mol/L in 0.1 mol/L Tris, pH 8.3) (Fluka) and 40 *μ*L HCY (0.75 mol/L in water) (Sigma‐Aldrich) solutions were added, mixed and incubated for 60 min at 37°C. The reaction was stopped with 20 *μ*L of 50% trichloroacetic acid (Merck). Samples were centrifuged and pellets discarded. 100 *μ*L supernatant was mixed with 825 *μ*L ninhydrin reagent [1 g ninhydrin (ACROS Organics), dissolved in 100 mL glacial acetic acid (Sigma‐Aldrich)mixed with 33 mL molten crystal phosphoric acid (Sigma‐Aldrich)]. The mixture of sample and reagent was incubated for 5 min in boiling water followed by 2 min on ice and 20 min at room temperature. The optical density of the resulting solution (455 nm) was measured against a blank sample. All samples were assayed in duplicate and CBS activity was calculated using a calibration curve generated with cystathionine (2–10 mmol/L) (Sigma‐Aldrich).

### Western blot analysis

Isolated tissues were homogenized in ice‐cold isolation solution with cocktail. The protein concentration was determined by Coomassie reagent. Protein lysates were denatured at 100°C for 10 min, separated by 12% SDS‐polyacrylamide gel electrophoresis, and transferred onto PVDF membranes. The bolts were blocked with 5% nonfat dry milk for 1 h and then probed with primary antibodies directed to CBS (Santa Cruz), CSE (Abcam) or *β*‐actin (Sigma‐Aldrich) (1:1000 dilution) overnight at 4°C, washed three times with Tris‐buffered saline (TBS) containing 0.1% v/v Tween‐20 and incubated for 1 h at room temperature (RT) with secondary antibodies (goat anti‐rabbit IgG and goat anti‐mouse IgG, Santa Cruz). The immunoreactive bands were visualized using chemiluminescent reagent (Thermo Scientific) and exposed to X‐ray film. Resulting blots were scanned and quantified using Image‐Pro Plus 6.0 software.

### Quantitative real‐time PCR

Total RNA was isolated using TRIzol (Invitrogen) and first‐strand cDNAs were synthesized from 4 *μ*g of total RNAs in a 20 *μ*L reaction using Superscript (Invitrogen). The first‐strand cDNAs served as the template for quantitative PCR (qPCR) performed in the Applied Biosystems 7900 Real Time PCR System using SYBR green PCR reagent. Oligonucleotides were designed using Primer3 software (available at http://frodo.wi.mit.edu/primer3/) and the sequences are listed as follows: *CBS* sense: 5′‐GGATTCCCCACATTACCACA‐3′ and antisense: 5′‐CTGATGCGGTCCTTCACAC‐3′; *CSE* sense: 5′‐ACCTTTGGCTCTGGGTGCT‐3′ and antisense: 5′‐TCCTGAAGTGTTTCTCCATCC‐3′; and *GAPDH* sense: 5′‐GTCTTCACTACCATGGAGAAGG‐3′ and antisense: 5′‐TCATGGATGACCTTGGCCAG‐3′. Cycling conditions were 95°C for 10 min, followed by 40 repeats of 95°C for 15 sec, and 60°C for 1 min.

### Immunohistochemistry

Kidneys were fixed with 10% formalin and embedded in paraffin. Kidney sections (4 *μ*m thickness) were incubated in 3% H_2_O_2_ for 15 min at room temperature to block endogenous peroxidase activity. After boiling in antigen retrieval solution (1 mmol/L tris‐HCl, 0.1 mmol/L EDTA, pH = 8.0) for 15 min at high power in a microwave oven, the sections were incubated overnight at 4°C with rabbit anti‐CBS antibody (Santa Cruz). After washing with PBS, the secondary antibody was applied and the signal was visualized using an ABC kit (Santa Cruz Biotechnology). The expression was quantified in the entire area of each sample in a semi‐quantitative fashion. The expression was ranked as follows: 0 in case of absence of staining or staining in less than 10% of cells; 1 for staining in 10% to 30% of cells; 2 for staining in 31–60% of cells; and 3 for staining in more than 60% of cells. The categories were then averaged to facilitate statistical analyses (Nishimura et al. 1996; Wilson et al. 2017).

### Statistical analysis

All values are presented as mean ± SE. Statistical analysis was performed using a Student's *t* test. Differences were considered to be significant when *P *< 0.05.

## Results

### Downregulation of CBS expression in the kidney in HFD mice

**Figure 1:**
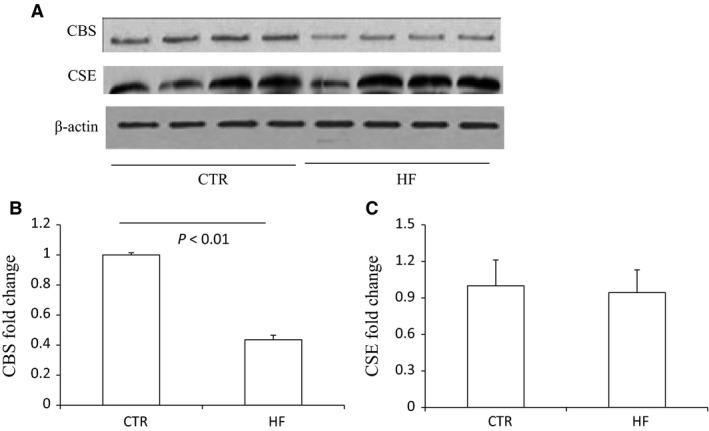
Effects of HFD on the protein expression of CBS and CSE in the kidney. (A) Western blot analyses of CBS, CSE, and beta‐actin. (B) Densitometry of CBS. A densitometric ratio between the densitometry of CBS and beta‐actin was calculated, and data are expressed in comparison with the controls. (C) Densitometry of CSE. A densitometric ratio between the densitometry of CSE and beta‐actin was calculated, and data are expressed in comparison with the controls. CTR, Control, *n* = 4; HF, High‐fat diet, *n* = 4. Data are means ± SE.

**Figure 2:**
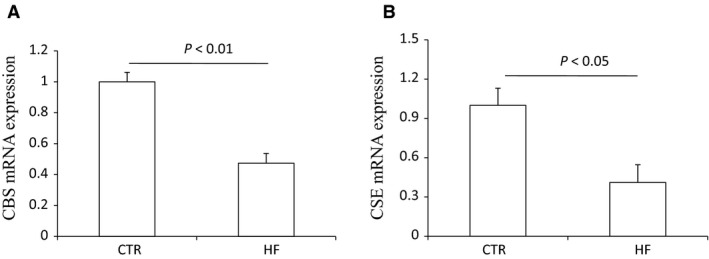
Effects of HFD on the mRNA expression of CBS and CSE in the kidney. (A) qRT‐PCR of analysis of CBS. (B) qRT‐PCR of CSE analysis. CTR, Control, *n* = 4; HF, High‐fat diet, *n* = 4. Data are means ± SE.

**Figure 3:**
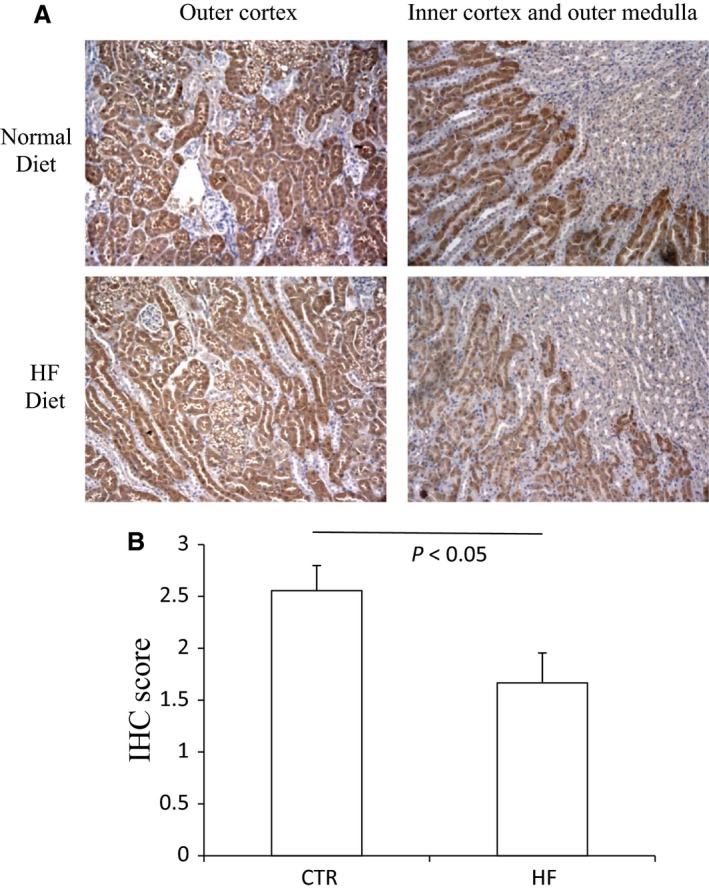
Representative immunohistochemical staining of CBS in renal tissues both after normal diet and high‐fat diet. (A) immunohistochemical staining of CBS. (B) immunohistochemical score of CBS.

**Figure 4:**
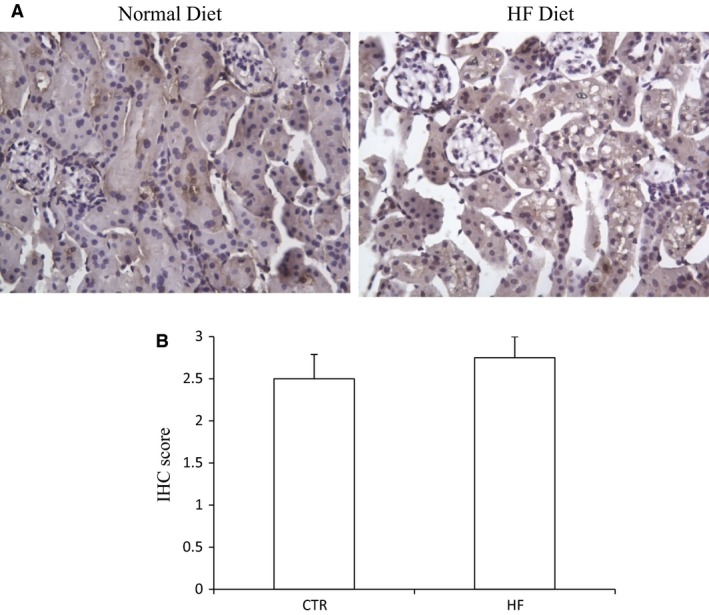
Representative immunohistochemical staining of CSE in renal tissues both after normal diet and high‐fat diet. (A) immunohistochemical staining of CSE. (B) immunohistochemical score of CSE.

**Figure 5:**
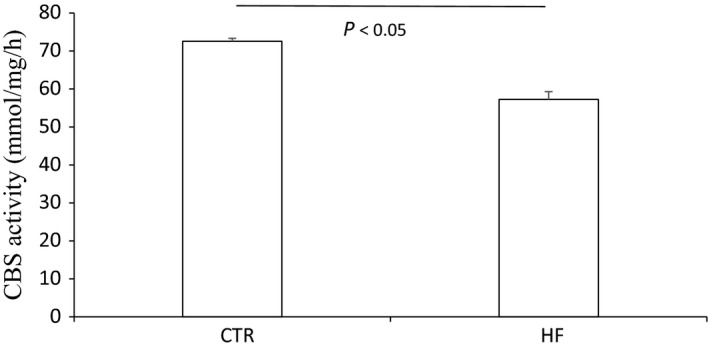
CBS activity in the kidney both after normal diet and high‐fat diet.

The body weight, plasma glucose, insulin, and triglyceride of control mice and HF mice are shown in Figure S1. The morphology was described as Figure S2. The renal CBS protein level in HFD mice was significantly decreased compared to control diet mice while CSE protein remained unchanged (Fig. 1). The expression of 3‐MST was neither changed after HF diet (Fig. S3). The mRNA expression of CBS in HFD mice was also down‐regulated although CSE mRNA was decreased at the same time (Fig. 2). The immunohistochemistry results confirmed the down‐regulation of CBS (Fig. 3) and unchanged expression of CSE (Fig. 4) in the kidney, which also showed that CBS and CSE was predominantly expressed in proximal tubules but not in glomeruli or distal tubules. The pattern of CBS localization in the kidney was consistent with a previous study (Yamamoto et al. 2013). Moreover, we measured CBS activity in renal homogenate. The standard curve was shown in Figure S4. Our data showed that renal CBS activity exhibited a significant decrease in HFD mice compared to control diet mice (Fig. 5). Taken together, CBS was specifically downregulated in the kidney during HFD.

### Specific decreased CBS expression in the kidney in db/db mice

**Figure 6:**
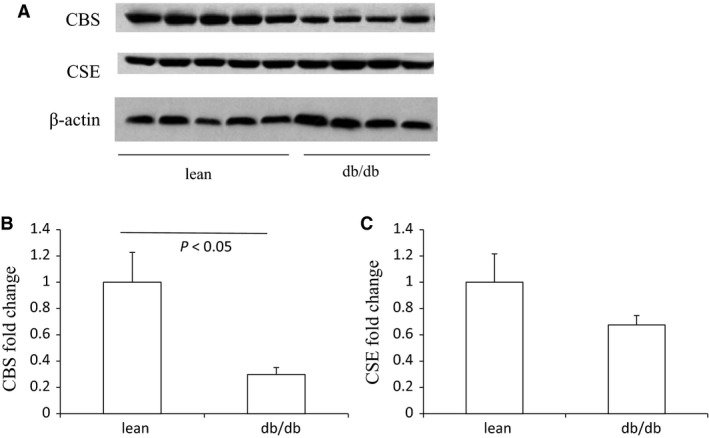
The protein expression of renal CBS and CSE in db/db mice and lean controls. (A) Western blot analyses of CBS, CSE, and beta‐actin in the kidney. (B) Densitometry of CBS. A densitometric ratio between the densitometry of CBS and beta‐actin was calculated, and data are expressed in comparison with the controls. (C) Densitometry of CSE. A densitometric ratio between the densitometry of CSE and beta‐actin was calculated, and data are expressed in comparison with the controls. lean, lean mice, *n* = 5; db/db, db/db mice, *n* = 4. Data are means ± SE.

**Figure 7:**
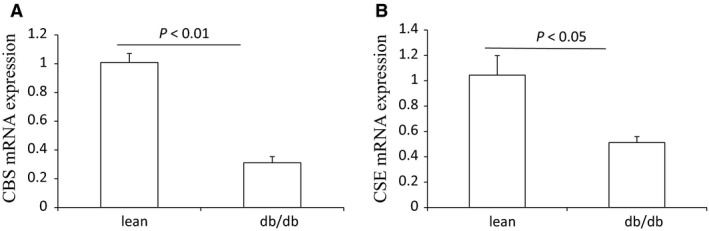
The mRNA levels of renal CBS and CSE in db/db mice and lean controls. (A) qRT‐PCR of analysis of CBS. (B) qRT‐PCR of CSE analysis. lean, lean mice, *n* = 5; db/db, db/db mice, *n* = 4. Data are means ± SE.

The body weight, plasma glucose, BUN, and creatinine of lean control mice and db/db mice are shown in Figure S5. Similar to the pattern in HFD mice, the renal expression of CBS protein showed an obvious decrease in db/db mice compared with lean control mice, and at the same time CSE expression was not changed (Fig. 6). The mRNA level of renal CBS was also reduced in db/db mice (Fig. 7). Different from the protein expression, the mRNA level of CSE in the kidney was decreased in db/db mice. Taken together, CBS expression was selectively decreased in the kidney both in HFD mice and gene mutation‐induced obese mice.

### Different pattern of expression of CBS and CSE in other tissues

**Figure 8:**
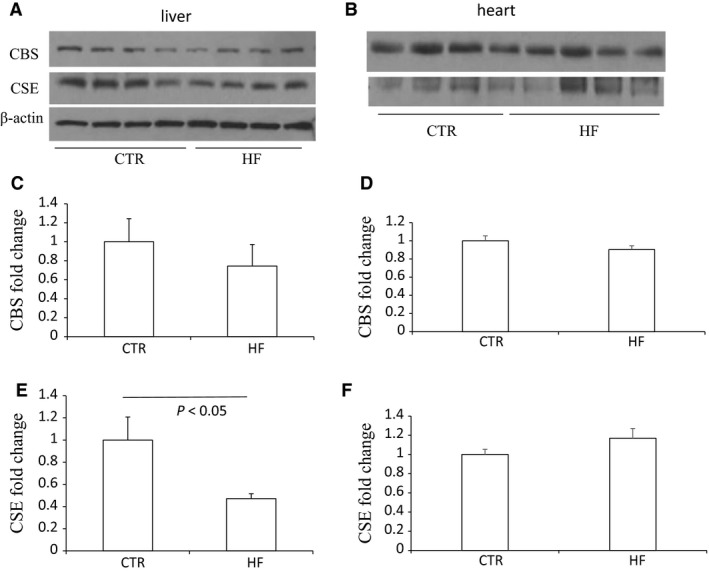
Effects of HFD on the protein expression of CBS and CSE in the liver and heart. (A–B) Western blot analyses of CBS, CSE and beta‐actin in the liver (A) and heart (B). (C and E) Densitometry of CBSand CSE in the liver. A densitometric ratio between the densitometry of CBS/CSE and beta‐actin was calculated, and data are expressed in comparison with the controls. (D and F) Densitometry of CBS and CSE in the heart. The densitometry of CBS and CSE was normalized by the bands of ponceau staining, and data are expressed in comparison with the controls. CTR, Control, *n* = 4; HF, High‐fat diet, *n* = 4. Data are means ± SE.

**Figure 9:**
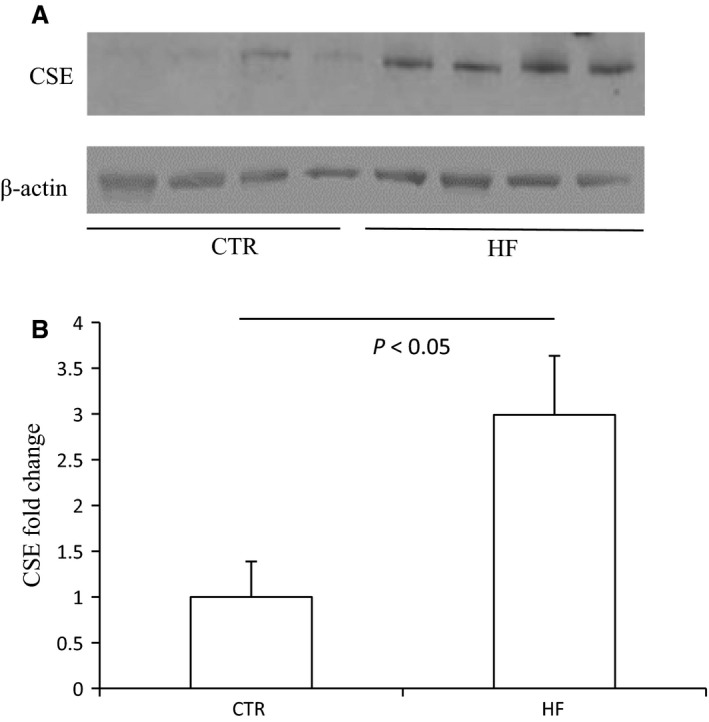
Effects of HFD on the protein expression of CSE and GLUT4 in epididymal fat. (A) Western blot analyses of CSE, GLUT4 and beta‐actin. (B) Densitometry of CSE. A densitometric ratio between the densitometry of CSE and beta‐actin was calculated, and data are expressed in comparison with the controls. (C) Densitometry of GLUT4. A densitometric ratio between the densitometry of GLUT4 and beta‐actin was calculated, and data are expressed in comparison with the controls. CTR, Control, *n* = 4; HF, High‐fat diet, *n* = 4. Data are means ± SE.

To further confirm the specificity of renal CBS downregulation after HFD, we monitored the protein levels of these two enzymes in other tissues including liver, heart and epididymal fat. According to the results shown in Figure 8, neither the level of CBS protein in the liver nor heart was changed by HFD, while CSE protein was decreased in the liver accompanied with unchanged expression in the heart. Since the main pathway of H_2_S generation in adipose tissues was mediated by CSE (Feng et al. 2009), we just detected CSE expression in epididymal fat. In contrast, it was strikingly increased after HFD (Fig. 9). To sum it up, the expression pattern of CBS and CSE was tissue‐dependent after HFD.

## Discussion

Feeding C57/BL6 mice with a high‐fat diet could induce obesity, insulin resistance, hyperlipidemia, hyperinsulinemia, hyperleptinemia, and excess circulating free fatty acid (Harte et al. 1999), which is a robust and efficient model for early type 2 diabetes and thus used as a tool for developing new potential therapeutic targets (Winzell and Ahren 2004). In the present study, we employed this model and demonstrated that CBS expression was selectively decreased in the kidney while CSE expression increased in epididymal fat. Also we confirmed the specific renal CBS downregulation in db/db mice model.

In the kidney, both CBS and CSE are responsible for the desulfuration of cysteine, leading to synthesis of H_2_S. Although CBS protein levels are 20‐fold lower than CSE in kidney, CBS constitutes the major source of H_2_S since propargylglycine (PAG), a specific inhibitor of CSE, adding to kidney extract reduced H_2_S production only by 15% under saturating substrate concentrations (Kabil et al. 2011). The current study showed decreased CBS in protein and activity levels while CSE expression remained unchanged in both diet‐induced and gene‐induced obese mice. Our study is limited in that we did not measure the H_2_S production in tissues. As shown in the literature, the downregulation of renal CBS/H_2_S pathway was also involved in both ischemia reperfusion (Xu et al. 2009) and Dahl‐salt‐sensitive hypertension (Huang et al. 2015). Moreover, renal H_2_S‐producing enzymes and capacity was significantly reduced during chronic kidney disease (CKD) (Aminzadeh and Vaziri 2012). Peh et al. (2014) reported that H_2_S production in kidney was reduced after HFD for 12 or 16 weeks despite unchanged CBS, CSE or 3‐MST protein expression. By the same token, CBS down regulation in the kidney might contribute to H_2_S deficiency, which resulted in the associated complications during obesity.

The pattern of the expression and regulation of CBS and CSE in the liver is different from that in the kidney. Under cellular conditions, CBS was estimated to account for only 3% of hepatic H_2_S generation (Kabil et al. 2011). We showed decreased CSE expression but unchanged CBS in the liver. Hwang et al. (2013) reported opposite results that HFD for 5 weeks stimulated hepatic CBS and CSE expression and also H_2_S production. In contrast, Peh et al. (2014) demonstrated consistent pattern with us that CSE expression in the liver was decreased in mice fed HFD for 8 and 16 weeks. It is possible that the up‐regulation of CSE/H_2_S pathway played an adaptive protective role during short‐term HFD while the down‐regulation acted as a pathogenic factor during chronic HFD.

Chronic imbalance of calories consumed versus expended causes increased intercellular triglyceride stored in adipose tissues characterized by adipose tissue expansion (Cai et al. 1861). Excess adipocyte differentiation (hyperplasia) and large adipocyte size (hypertrophy) or dysfunction of adipose lipolysis contributed to pathogenesis of obesity. We revealed that CSE was increased in epididymal fat after HFD. CSE/H_2_S was reported to induce adipogenesis in 3T3L1 cells (Tsai et al. 2015). Moreover, H_2_S donor reduced lipolysis while the CSE inhibitor PAG significantly increased it in adipocytes, and PAG blocked fat mass increase by increasing adipose lipolysis, thus attenuating insulin resistance after HFD (Geng et al. 2013). All these studies implied that CSE/H_2_S pathway in adipose tissues might promote adipogenesis. So the up‐regulation of CSE in epididymal fat probably was a pathogenic reason for increased fat mass and after HFD. Surprisingly, in the supplemental data of the above study, endogenous CSE expression was shown to decrease in epididymal fat (Geng et al. 2013). It is difficult to explain why PAG attenuated obesity after HF at the same time endogenous CSE was down‐regulated. It is possible that different source of animals, the composition of HFD or the duration of the study period resulted in the discrepancies between these two results.

In conclusion, this study for the first time reports the distinct regulation of H2S synthesizing enzymes including CBS and CSE during metabolic stresses. Renal CBS expression was consistently downregulated in DIO and db/db mice contrasting to up‐regulation of epididymal fat CSE in the DIO model. In the kidney, the suppressed CBS may contribute to reduced H_2_S production resulting in associated complications during obesity, which suggest a new potential therapeutic target of obesity. In epididymal fat, the activation of CSE/H_2_S pathway might decrease glucose uptake resulting in insulin resistance during obesity. Together, H_2_S display pleiotropic effects by different synthesis enzymes in different tissues, which might provide novel diagnostic marker or therapeutic target for clinical diseases and thus warrants further intensive study.

## Conflict of Interests

The authors have declared that no conflict of interests exists.

## Data Accessibility

## Supporting information




**Figure S1.** Body weight (A), plasma glucose (B), insulin (C) and triglyceride (D) after high‐fat diet. CTR, Control, *n* = 4; HF, High‐fat diet, *n* = 4. Data are means ± SE.
**Figure S2.** Representative images of periodic acid‐Schiff staining of kidneys after high‐fat diet. CTR, Control, *n* = 4; HF, High‐fat diet, *n* = 4.
**Figure S3.** Effects of HFD on the protein expression of 3‐MST in the kidney. (A) Western blot analyses of 3‐MST and beta‐actin. (B) Densitometry of 3‐MST. A densitometric ratio between the densitometry of 3‐MST and beta‐actin was calculated, and data are expressed in comparison with the controls. CTR, Control, *n* = 3; HF, High‐fat diet, *n* = 3. Data are means ± SE.
**Figure S4.** The standard curve of CBS activity measurement with ninhydrin method. (A) The absorbance OD value is linear to the concentration of cystathionine. (B) The CBS activity is linear to the protein amount of homogenate.
**Figure S5.** Body weight (A), plasma glucose (B), BUN (C) and creatinine (D) in db/db mice and lean controls. lean, lean mice, *n* = 5; db/db, db/db mice, *n* = 4. Data are means ± SE.
