# Supplementary material for: Specific downregulation of cystathionine β‐synthase expression in the kidney during obesity
Source: Physiol Rep. 2018 Jul 12;6(13):e13630. doi: 10.14814/phy2.13630 (PMC6041699; doi:10.14814/phy2.13630)
Supplement: Supplementary file 1 — Figure S1. Body weight (A), plasma glucose (B), insulin (C) and triglyceride (D) after high‐fat diet. CTR, Control, n = 4; HF, High‐fat diet, n = 4. Data are means ± SE. Figure S2. Representative images of periodic acid‐Schiff staining of kidneys after high‐fat diet. CTR, Control, n = 4; HF, High‐fat diet, n = 4. Figure S3. Effects of HFD on the protein expression of 3‐MST in the kidney. (A) Western blot analyses of 3‐MST and beta‐actin. (B) Densitometry of 3‐MST. A densitometric ratio between the densitometry of 3‐MST and beta‐actin was calculated, and data are expressed in comparison with the controls. CTR, Control, n = 3; HF, High‐fat diet, n = 3. Data are means ± SE. Figure S4. The standard curve of CBS activity measurement with ninhydrin method. (A) The absorbance OD value is linear to the concentration of cystathionine. (B) The CBS activity is linear to the protein amount of homogenate. Figure S5. Body weight (A), plasma glucose (B), BUN (C) and creatinine (D) in db/db mice and lean controls. lean, lean mice, n = 5; db/db, db/db mice, n = 4. Data are means ± SE. [file PHY2-6-e13630-s001.ppt]

## Slide 1
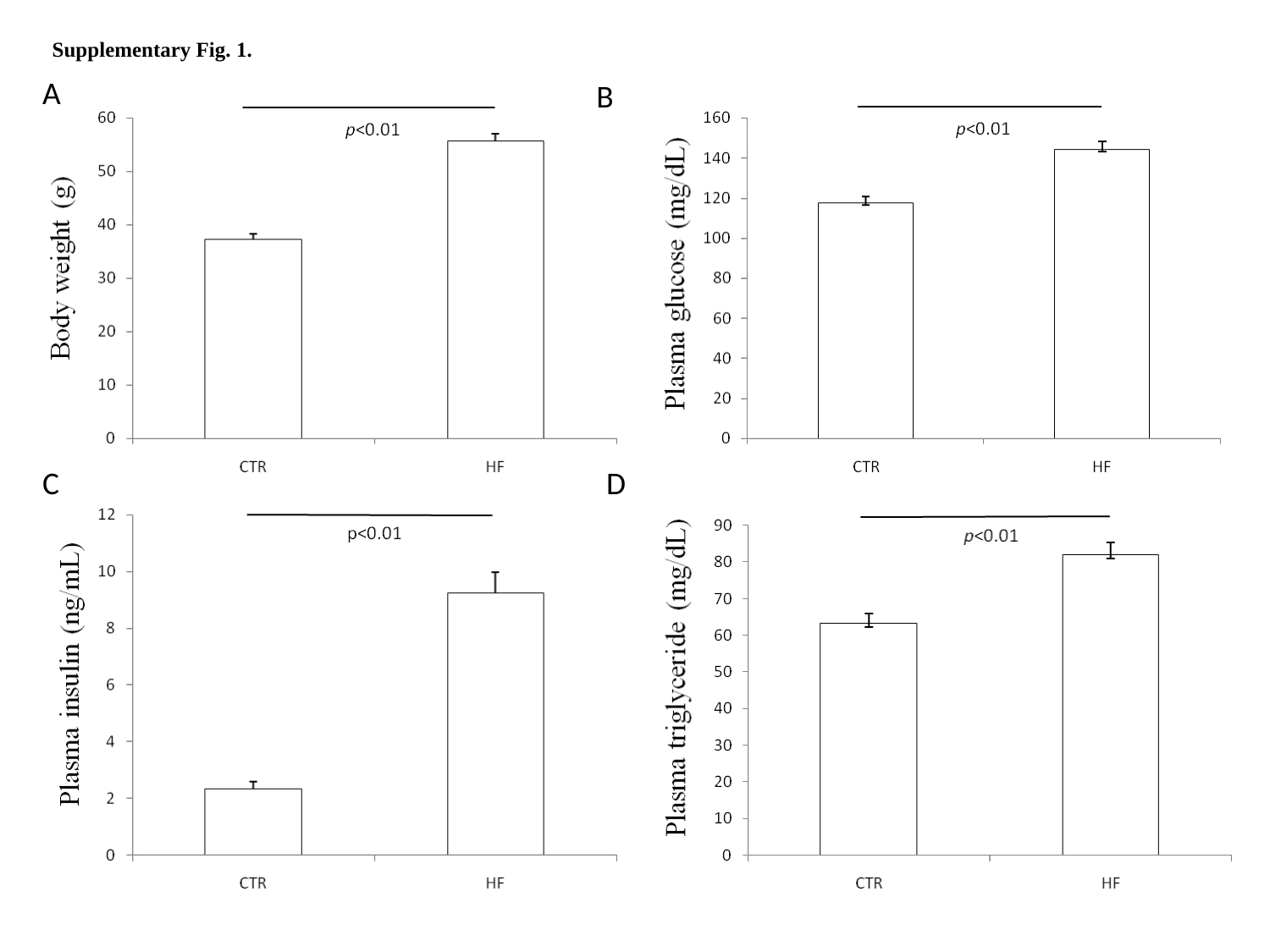

Supplementary Fig. 1.
A
B
C
D

## Slide 2
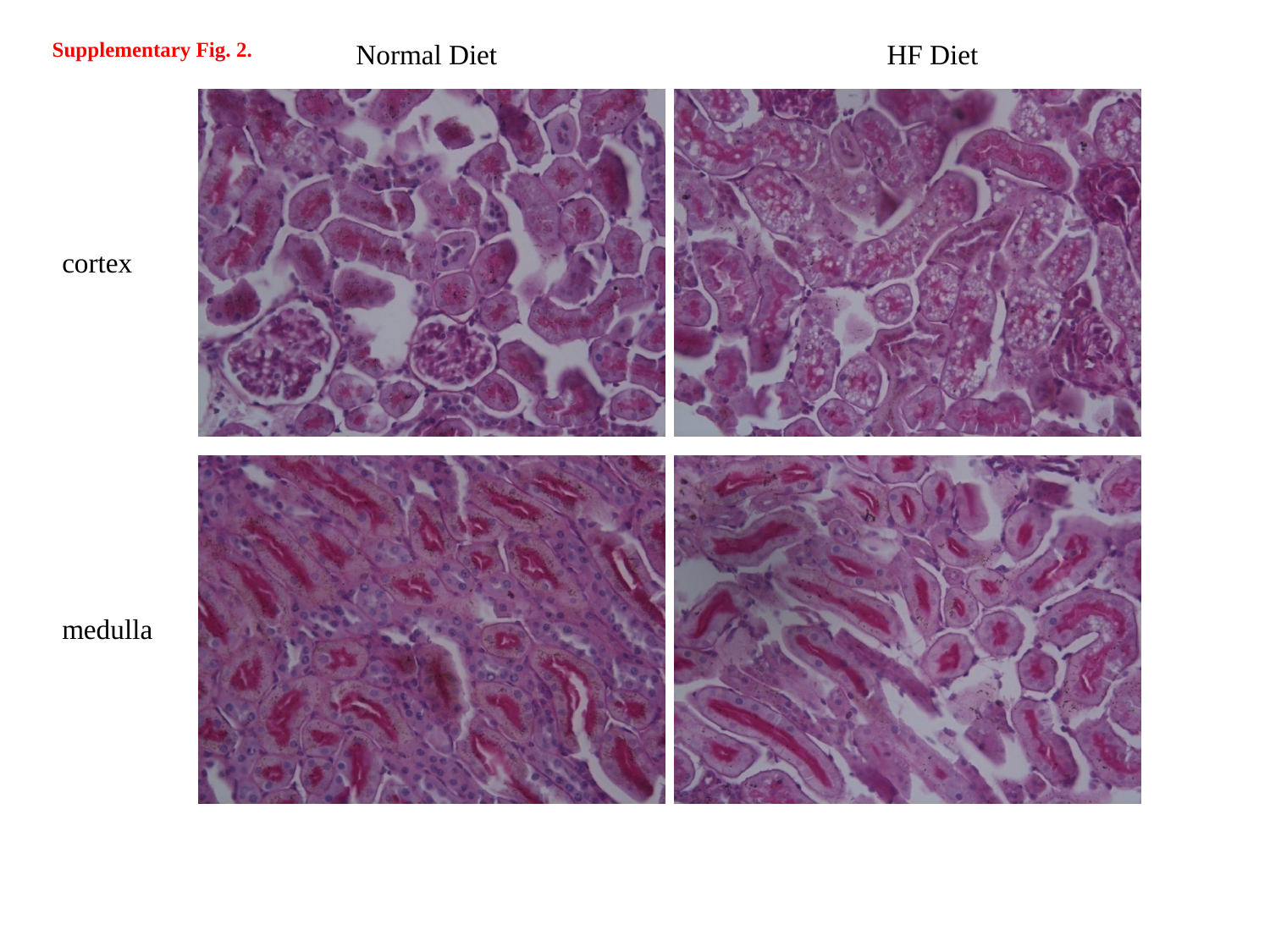

Supplementary Fig. 2.
Normal Diet
HF Diet
cortex
medulla

## Slide 3
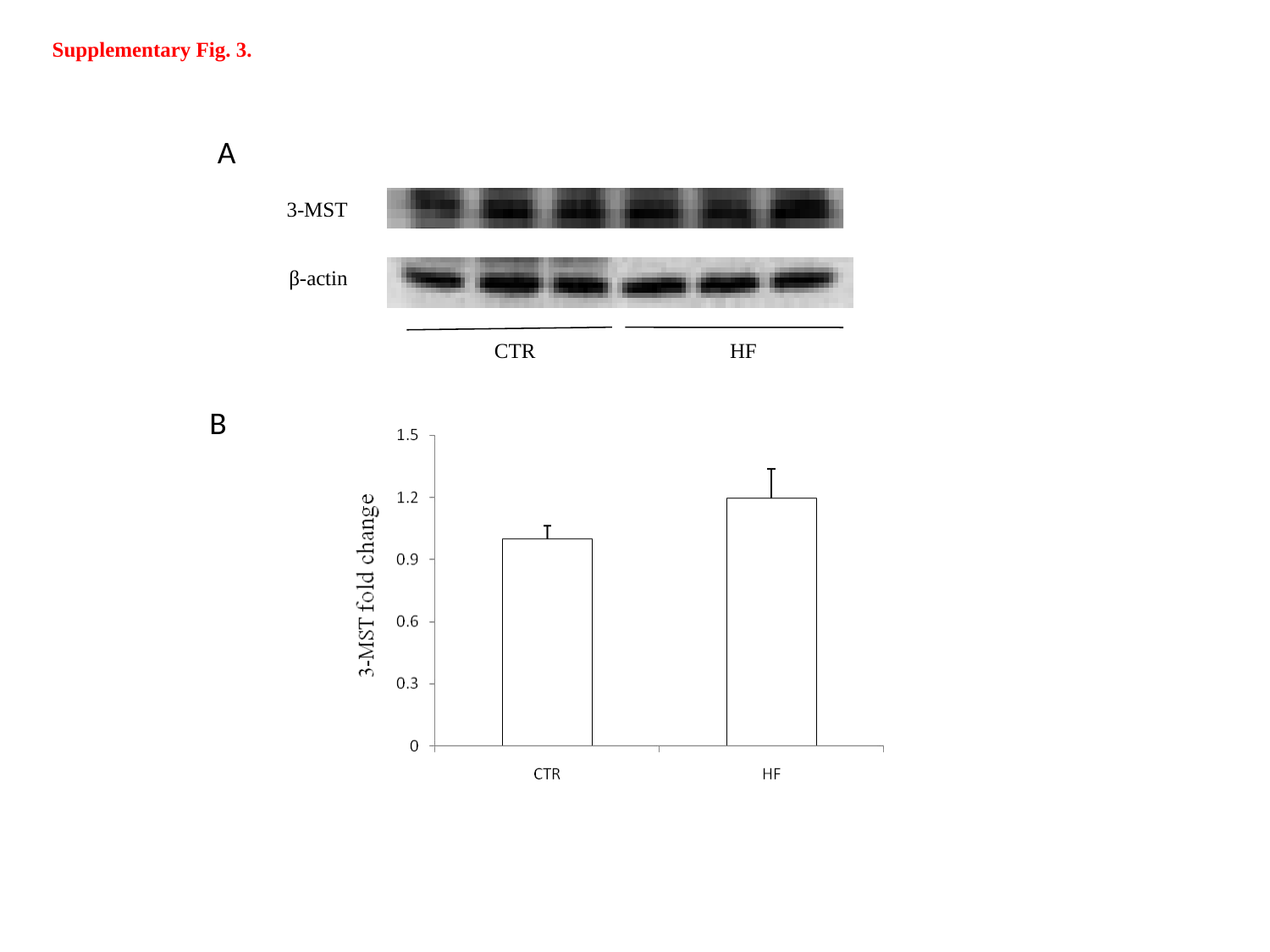

Supplementary Fig. 3.
A
3-MST
β-actin
CTR
HF
B

## Slide 4
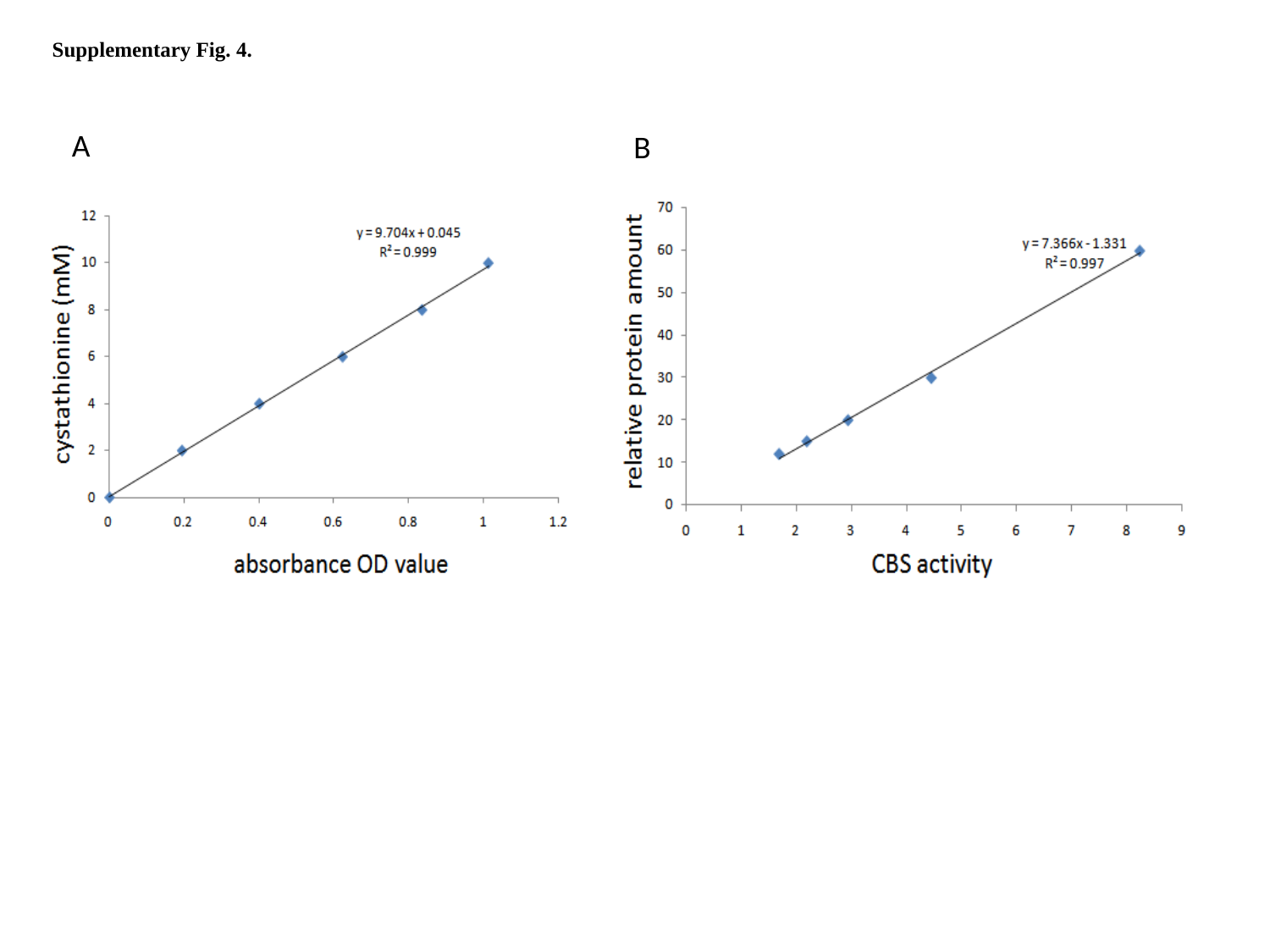

Supplementary Fig. 4.
A
B

## Slide 5
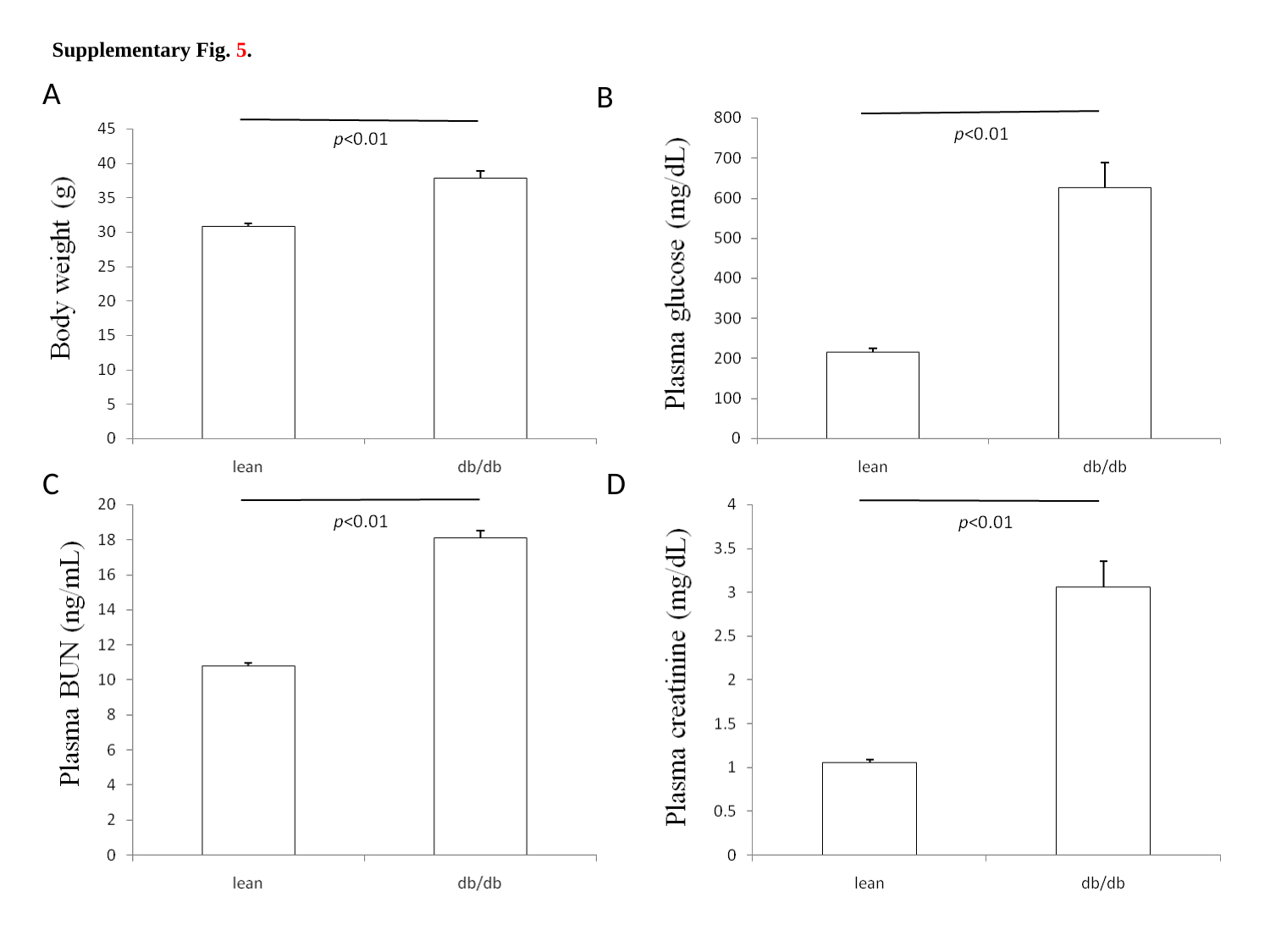

Supplementary Fig. 5.
A
B
C
D
